# Cell fishing: A similarity based approach and machine learning strategy for multiple cell lines-compound sensitivity prediction

**DOI:** 10.1371/journal.pone.0223276

**Published:** 2019-10-07

**Authors:** E. Tejera, I. Carrera, Karina Jimenes-Vargas, V. Armijos-Jaramillo, A. Sánchez-Rodríguez, M. Cruz-Monteagudo, Y. Perez-Castillo

**Affiliations:** 1 Ingeniería en Biotecnología, Facultad de Ingeniería y Ciencias Aplicadas, Universidad de Las Américas, Quito, Ecuador; 2 Grupo de Bio-Quimioinformática, Universidad de Las Américas, Quito, Ecuador; 3 Departamento de Informática y Ciencias de la Computación, Escuela Politécnica Nacional, Quito, Ecuador; 4 Departamento de Ciências de Computadores, Faculdade de Ciências, Universidade do Porto, Porto, Portugal; 5 Universidad Técnica Particular de Loja, Loja, Ecuador; 6 Center for Computational Science (CCS), University of Miami (UM), Miami, FL, United States of America; 7 West Coast University, Miami, Florida, United States of America; 8 Escuela de Ciencias Físicas y Matemáticas, Universidad de Las Américas, Quito, Ecuador; University of Birmingham, UNITED KINGDOM

## Abstract

The prediction of cell-lines sensitivity to a given set of compounds is a very important factor in the optimization of in-vitro assays. To date, the most common prediction strategies are based upon machine learning or other quantitative structure-activity relationships (QSAR) based approaches. In the present research, we propose and discuss a straightforward strategy not based on any learning modelling but exclusively relying upon the chemical similarity of a query compound to reference compounds with annotated activity against cell lines. We also compare the performance of the proposed method to machine learning predictions on the same problem. A curated database of compounds-cell lines associations derived from ChemBL version 22 was created for algorithm construction and cross-validation. Validation was done using 10-fold cross-validation and testing the models on new data obtained from ChemBL version 25. In terms of accuracy, both methods perform similarly with values around 0.65 across 750 cell lines in 10-fold cross-validation experiments. By combining both methods it is possible to achieve 66% of correct classification rate in more than 26000 newly reported interactions comprising 11000 new compounds. A Web Service implementing the described approaches (both similarity and machine learning based models) is freely available at: http://bioquimio.udla.edu.ec/cellfishing.

## Introduction

The prediction of a cell-line sensitivity to chemical compounds is a very important factor to optimize *in-vitro* assays in the drug discovery processes. The usual prediction approaches addressing this problem can be divided into two major groups:

- Approaches based on quantitative structure-activity relationships (Q)SAR modeling. These approaches aim at establishing a model relating chemical structures with their effect on different cell lines [1–6].
- Approaches based on gene expression such as the well-known CMAP and L1000CDS2 methodologies [7–9]. Other methods had been explored in this type of strategies but all of them rely on the CMAP or L1000 projects [10].

The goal of the methods using gene expression is to compare the genetic expression profile triggered by a query compound with a known reference expression pattern generated with a particular cell line-compound pair. Therefore, these methods require the gene expression profile triggered by the compounds under investigation, which is frequently unknown.

On the other hand, QSAR strategies had been previously applied for drugs-cell lines sensitivity prediction [1–6] with satisfactory results. All these studies used some type of modeling involving optimization functions (random forest, SVM, kernel optimization, etc) and in several cases the similarity between cell lines is also included in the model (i.e. genome expression profile, mutations in cell lines, etc) [2,5]. On the other hand, the number of cell lines and/or compounds used in previous studies is sometimes reduced in order to fulfill the models criteria. For example, Ammad-ud-din et al [5] modelled 650 cancer cell lines but employing only 116 compounds while Lagunin et al [1] studied 59882 compounds and 278 cancer cell lines.

The core idea behind any QSAR model is the similarity principle. This principle postulates that compounds sharing a high structural similarity should also have similar activities/properties. Actually, the work of Zhang et al [6] clearly corroborate that similar compounds induce similar cells response in terms of gene expression for more than 120 drugs and 600 cancer cell lines. However, the similarity principle is sometimes disrupted and depends on the corresponding structure-property space. Such space is determined by the chemical description used (i.e. chemical fingerprints, molecular descriptors) and the associated properties (i.e. the biological property of interest) [11,12]. The modeling task attempts to discover the best function capable of representing the structure-property space. In this sense, even when several mathematical approaches had been explored, machine learning is by far the most used strategy in QSAR modeling. A comprehensive review of Ruolan et al cover several strategies specially focused on machine learning and similarity [13]

In the present work, we went through a direct similarity-based strategy being the first time that this approach is evaluated for predictions of compounds-cell lines interactions. Under this approach, no learning based algorithm is used to model the structure-activity space and only the consideration of molecular similarity is required. It is a simple and fast approach that was first applied in the area of chemicals-proteins interactions prediction and later coined as “target fishing” [14–17]. In this sense, a similarity-based strategy is not only the simplest one but also has the potential to be easily extended to different cell lines without the need of highly curated databases [18,19]. These features are desirable with the constantly growing amount of data in databases such as ChemBL [20] and PubChem [21]. Our goal is to explore the performance of a similarity-based approach for the prediction of potential compounds-cell lines interactions and compare it with machine learning algorithms evaluated on the same problem.

## Results and discussion

### Overall performance of the cell fishing approach

**Fig 1:**
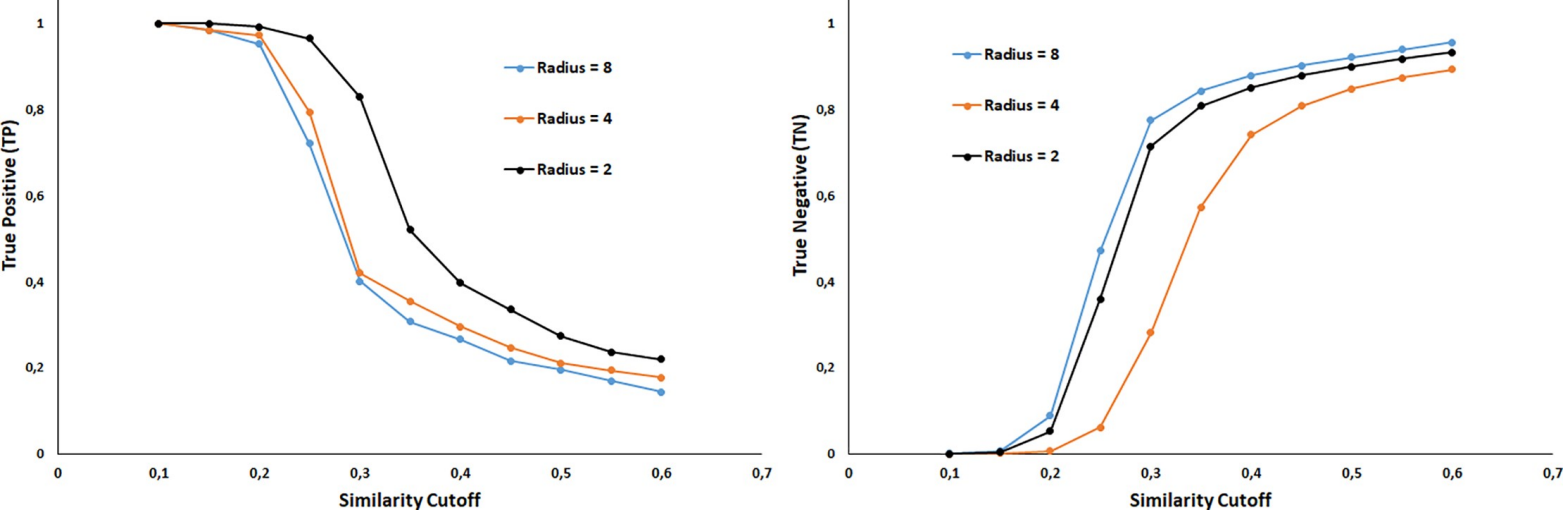
Variation in the true positive (TP) (Left) and true negative (TN) (Right) with respect to similarity cutoff considering Morgan radius of 2, 4 and 8. Results obtained from 10-fold cross-validation.

**Fig 2:**
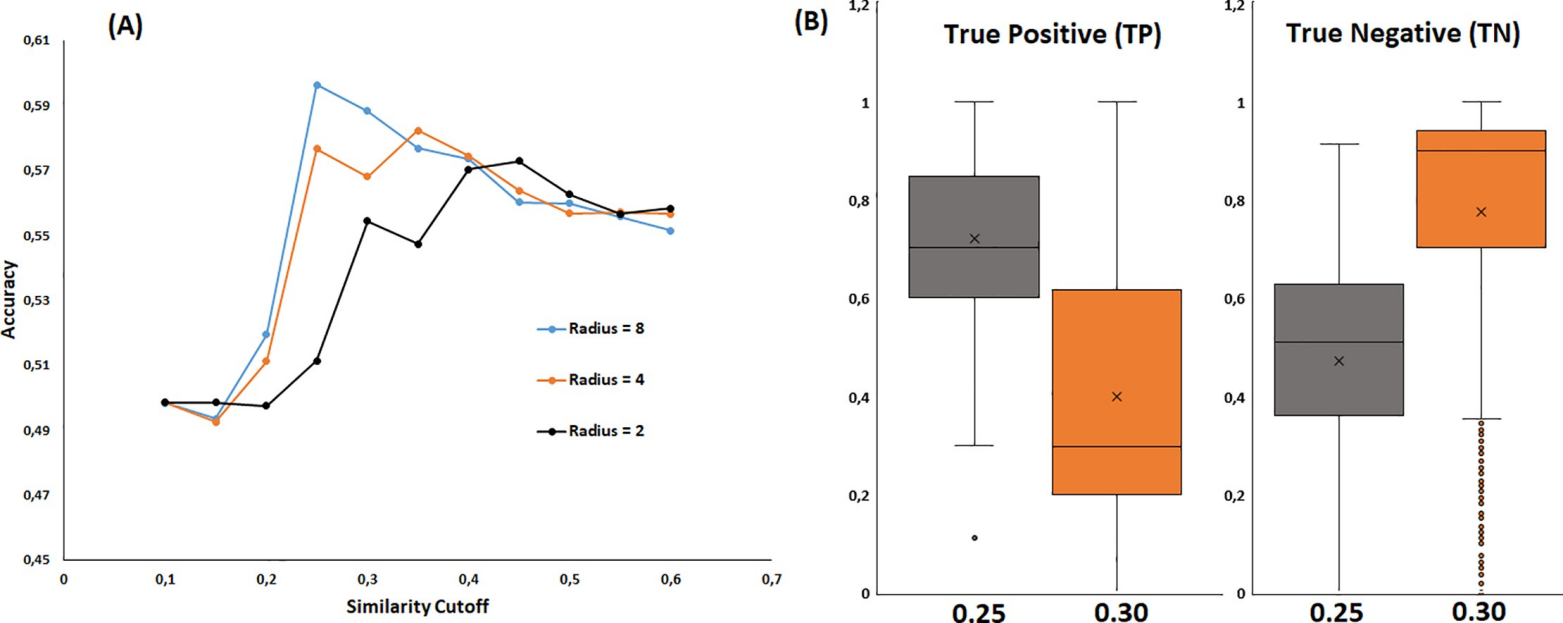
Left (A) Variation in the accuracy (ACU) with respect to similarity cutoff considering Morgan radius of 2, 4 and 8. Right (B) Boxplot for true positive (TP) and true negative (TN) in 758 cell lines for similarity cutoff of 0.25 and 0.30 with Radius = 8.

We first evaluated the influence of the radius of the MCFPs and similarity cutoff on the performance of our “cell fishing” or similarity-based approach. The results of these analyses are summarized in Fig 1. Increasing the similarity cutoff decreases the true positives rate and increases the true negative detection. This means that selecting higher similarity values increases the stringency of the model, yielding fewer sensible interactions but with highest confidence. Increasing the Morgan radius in the fingerprint computation increases both resolution and predictability. The effect of the Morgan radius on the prediction metrics is shown in Fig 1, however, it is clearest seen in Fig 2(A) were the choice of Radius = 8 leads toward maximum accuracy in the similarity interval 0.2–0.3.

In Fig 2(B) it can be noticed that, with Radius = 8, for similarity cutoff of 0.25 around 50% of the data show TP > 0.65 and for around 25% of the data TP> 0.8 with TN>0.6. This is a clear sign that in many cell lines the similarity-based strategy (also machine learning, as we will see next) is a good prediction strategy. Actually, the ratio of cell lines with good predictions is similar to those obtained by Lagunin et al [1] using a machine learning strategy. These authors initially started with 943 human cell lines and 59882 compounds but the final model was only reported for the cell lines with AUC higher than 0.8 (278 cancer cell lines, 29.4%).

The similarity-based strategy compares a query compound with known sensitive interaction for a particular cell lines. Therefore, the goal is to detect sensitive response. Increasing the similarity cutoff reduces the chances of finding similar molecules but if detected, the confidence will be higher (Figs 1 and 2). Accuracy values (Fig 2) are lower than those obtained in target modelling [14–17,22]. The inhibition of a cell line growth can be accomplished through several known and unknown mechanisms usually involving different targets. In contrast, targets ligands can be reduced to a few chemical scaffolds. Therefore, the chemical diversity is larges in cell line-compound problems compared with the chemical diversity found in a compound-target prediction.

### Comparison between the similarity-based approach and machine learning

As described in the methodology section, for SVM we choose cell lines with more than 20 compounds with reported sensitive and resistant interactions (a minimum of 40 compound per cell line). However, for the similarity-based approach we only need 10 sensitive compounds. This difference reduces the cells space from 960 cell lines and 109349 compounds with 140114 sensitive and 147690 resistant LCLAs to 758 cell lines and 107195 compounds with 134565 sensitive and 145064 resistant LCLAs.

**Fig 3:**
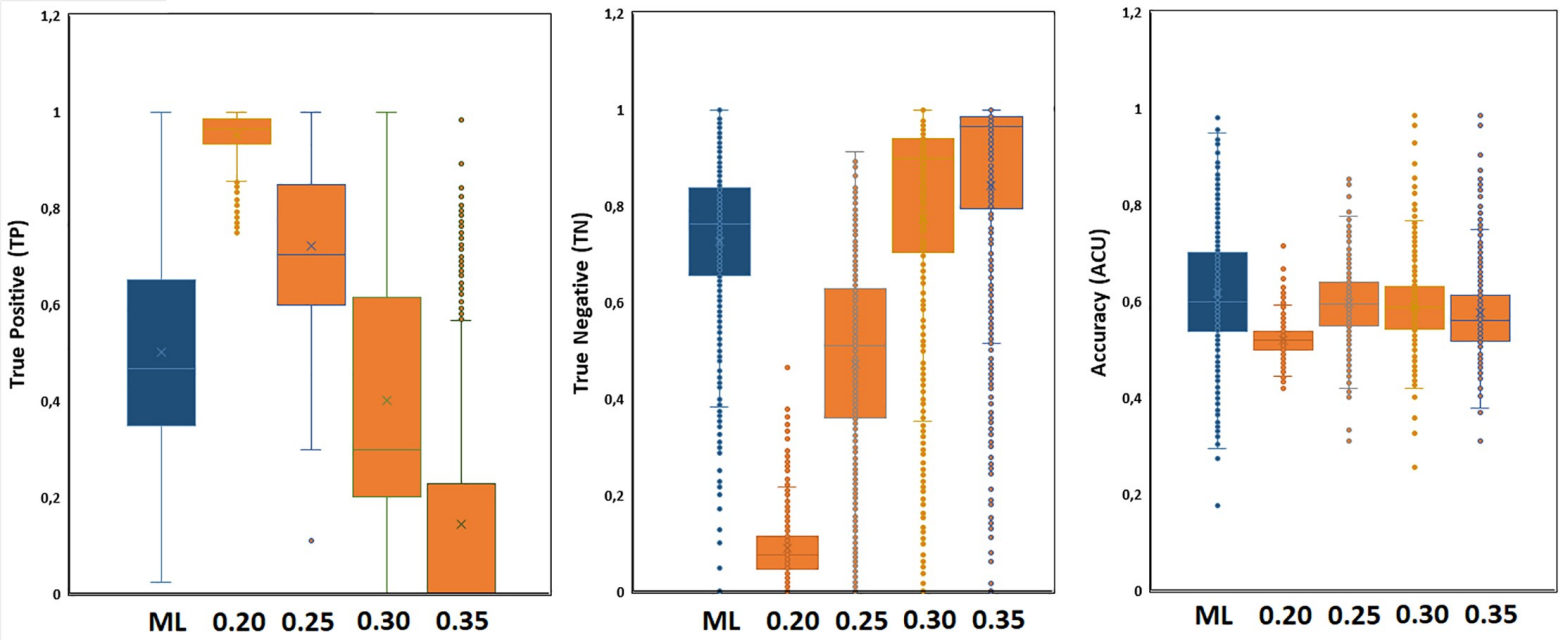
Comparative result in terms of accuracy, true positive and true negative, for SVM (blue boxes) and similarity-based strategy (orange boxes) under several cutoff similarities.

It can be noticed (Fig 3) that closer results between SVM model and similarity-based approach are obtained for similarities between 0.25–0.30. For a similarity cutoff of 0.25 cell fishing clearly tend to overestimate the sensitive associations compared to SVM. On the other hand, lower values of the similarity cutoff will lead to increase the false positives detection. However, for a cutoff of 0.3, 50% of the predicted true positive responses already contain 75% of the predictions made with SVM with higher specificity regarding true negative detection. It is possible that a middle value between 0.25–0.30 provides closest results to SVM in terms of TP and TN detection.

In general, some sensitive/resistant association are better predicted with the similarity approach and vice versa. Moreover, several cell lines are equally modelled with both methods (Table 1). By keeping the cell lines with accuracy higher than 0.65 and 0.7 (Table 1) according to both methods over 300 cell lines can be covered with true-positive and true-negative detection levels around 70% and 80%, respectively. These detection rates are higher in about 200 cell lines. These results are better than previous published methods considering the number of compounds-cell lines under study (see SM3).

**Table 1:** Performance indexes obtained in 10-fold cross-validation by combining both methods under cutoff distance of 0.25 and 0.3. Results are limited to cell lines with accuracy of 0.65 and 0.7.

	Accuracy (ACU)			
	0.65	0.65	0.7	0.7
Similarity Cutoff	0.25	0.3	0.25	0.3
Cell Lines (SVM)	258		189	
TP (SVM)	0.700		0.758	
TN (SVM)	0.82		0.830	
Cell Lines (Similarity)	162	123	48	54
TP (Similarity)	0.722	0.67	0.791	0.777
TN (Similarity)	0.661	0.75	0.699	0.750
Cell Lines (Common)	65	98	27	45
Cell Lines (Total)	355	283	210	198
TP (Total)	0.69	0.675	0.751	0.758
TN (Total)	0.786	0.832	0.825	0.834

### Exploring predictability in new molecules

The objective of both considered strategies is to identify the maximal amount of interactions between a query compound and all cell lines under consideration. Therefore, the goal in this experiment is to evaluate the capacity to identify all possible sensitive/resistant interactions for new molecules. In this sense, the metrics were calculated across the interactions space. This experiment is not comparable with cross-validation results because on the later the metrics were evaluated across cell lines. That is, in the 10-fold cross-validation the true positives ratio indicates the average detection across cell lines, instead of the true positive detection ratio in all known sensitive/resistant interactions for a given set of compounds (as in Table 2).

**Table 2:** Performance indexes across new compounds in external validation.

Model	Method	Compounds/ Interactions	Cell lines	TP	TN	Coverage^1^	TP_C^2^	TN_C^2^
1	SVM	11202 / 26135	758	0.610	0.663	100%		
2	Similarity-based (0.20)			0.572	0.574	91.76	0.617	0.551
3	Similarity-based (0.25)			0.376	0.765	65.5	0.550	0.673
4	Similarity-based (0.30)			0.260	0.843	47.56	0.515	0.722
5	Similarity-based (0.20) and SVM			0.617	0.551	100%		
6	Similarity-based (0.25) and SVM			0.661	0.700	100%		
7	Similarity-based (0.30) and SVM			0.523	0.656	100%		

Notes: 1) Coverage: the amount of compounds in which at least one prediction was made with similarity-based approach. 2) TP_C and TN_C are the true positive and true negative evaluated only in the portion of compounds covered by similarity-approach.

Three strategies were evaluated in external validation (Table 2):

1. The algorithm based on SVM over the 758 cell lines.
2. The similarity-based algorithm over the 758 cell lines using the similarity cutoff of 0.20, 0.25 and 0.3.
3. The combined SVM and similarity-based algorithms over the 758 cell lines using the similarity cutoff of 0.25.

The similarity-based approach apparently lead to poor prediction compared to SVM. However, it is important to remember that the similarity-based strategy is based on comparing only to similar sensitive compounds. Therefore, it is possible that some of the new compounds cannot be compared to any of the existing ones in the database.

For a cutoff of 0.20, the similarity approach covers almost 92% of the compounds, which means that for 10279 new compounds at least one cell line was predicted based on similarity. In this space, the true-positive and true negative ratios are comparable to those obtained by SVM. Actually, the inclusion of SVM models for the remaining 8% of the compounds do not improve performance (Table 2, model 5). Also, increasing the cutoff in the similarity-based model lead to a reduction in TP because of the reduction in the number of predicted cell lines. However, the considerable increment in TN indicates that the specificity of the obtained interaction increases. Using a higher similarity cutoff provides lower number of predicted cells but a higher confidence for those predicted cell lines.

The model 6 (Table 2) is the result of combining both strategies: we apply the similarity-approach using a similarity cutoff of 0.25 and in those compounds with no prediction (35.5%), we applied the SVM model. The result is better compared with any method separately.

### Advantage, limitations and further considerations

In the S3 Table we show a comparison between previous models and our current algorithm including cells and compounds diversity, source databases, modeling strategy and performance metric. Because of the highly heterogeneous data and modelling strategies is hard to stablish a direct comparison. Moreover, no software is available for prediction of several smiles at once in order to stablish a common benchmark.

The ChemBL database is continuously growing regarding the number of assays as well as the number of new compounds. The addition of new compounds and biological assays, if SVM models are used for LCLAs predictions, will require the reevaluation of the entire model so scalability will become an important issue. In a similarity-based strategy, this problem can be simply addressed. In both cases, the compounds additions will lead to improvements in predictability because they will necessarily increase the molecular as well as the cell lines diversity.

The machine learning strategy (as used in this work) provides several advantages including the learning-based extrapolation capabilities, which is noticed in the external data set evaluation. However, compared to the similarity approach, the latter is simplest and easily scalable. Even when both strategies are relatively close in terms of cross-validation, the evaluation of the external data indicates that new chemical scaffolds will be hard to predict using the similarity based strategy (coverage reduction). This is why better results were obtained combining both approaches. Our results also suggest that comparison, whenever possible, between advanced modelling techniques and the simpler strategies (in this case the direct similarity comparison) need to be considered.

The prediction of a particular property using the chemical similarity principle is sensible to the description space used for modelling. In this research, we rely upon a comparison based on Tanimoto similarity measured over Morgan fingerprints. If for a particular cell-line only a small and conserved group of molecules is available, then the similarity will be small for any other query with a new molecular scaffold. Hence, better models (using machine learning and/or similarity-based) could be obtained including other descriptions of the molecular space combined with feature selection algorithms.

## Materials and methods

### ChemBL database processing and curation

The ChemBL database version 22 [20] was filtered in order to obtain a curated database for ligand-cell line associations (LCLA), suitable for model fitting and validation. First, the *cell_dictionary* table distributed with ChemBL v22 in MySQL format was used to extract all cell identifiers (*cell_id*). Only those LCLA linked to assays reporting median inhibitory concentration (IC_50_), the median growth inhibitory concentration (GI_50_) and/or the median cytotoxic concentration (CC_50_) were considered for further processing. The canonical smiles of each compound was independently analyzed. In cases of salts or disconnected structures, only the largest fragment was used as the input structure. Next. LCLAs established at a ligand concentration lower than or equal to 10^−5^ M (inhibition of more than the 50% of cell growth at a concentration lower or equal than 10 μM) were labeled as “sensitive”, and the rest were labeled as “resistant”. When more than one assay is reported for the same LCLA, it is removed if there is no total agreement between assays (i.e. all assays must lead to the same sensitive or resistant classification). Finally, any cell line sensitive to less than 10 compounds was removed from the final database. The combination of several assay types (ex. IC_50_. GI_50_ and CC_50_) into a single classification rule has been recently used by other authors in compounds-cell-lines sensitivity prediction [1]. Moreover, it is a common strategy in the prediction of protein-compound interaction problems [14,15].

Accordingly, the final database included 960 cell lines comprising 140114 sensitive and 147690 resistant LCLAs. Details on the cell lines included in the final database can be found in the S1 and S2 Tables respectively.

### Similarity based prediction methodology

In this work. the target fishing approach proposed in Liu et al and recently applied by Peng et all [14,23] was adapted for cell line fishing. To be more precise, the implementation reported in [24] was adapted for cell line fishing by considering LCLAs instead of ligand-target interactions. Thus, our approach bases on ligand similarity, which assumes the similarity principle as the rationale to infer new LCLAs. In other words, structurally similar compounds should have identical associations with the same cell line. Therefore, the main purpose of our cell line fishing approach is to identify a set of cell lines that can establish sensitive associations with a query ligand.

To measure structural similarity, chemical structures were encoded using the Circular Morgan Fingerprints (CMFP), which are equivalent to the Extended Chemical Fingerprints (ECFP). implemented in RDKit [25]. The Tanimoto coefficient (Tc) was selected as the molecular similarity metric. Accordingly, a query ligand is considered to establish a sensitive association with a cell line if its average structural similarity with the top *n* closest sensitive compounds reported in the database for that cell line is higher than a certain similarity cutoff.

In this work, we tested n = 3 based on the average molecular similarity of the top 3 closest ligands. The efficiency of several similarity cutoffs for the CMFP defined by different radii in detecting sensitive associations was also explored. The evaluated similarity cutoffs ranged from 0.2 to 0.6 with a step size of 0.05. Three different radii of the CMFP were tested: *r* = 2 (ECFP4). r = 4 (ECFP8). and r = 8 (ECFP16). The effect on predictability of different similarity cutoffs were explored. However. we should keep in mind the standard of chemical similarity established for most cheminformatics applications: a pair of molecules is considered as structurally similar if they share an ECFP4 Tc equals or higher than 0.55 [26].

### Machine learning based methodology

Machine learning (ML) classification models were evaluated with 758 cell lines out of the initial pool of 960 cell lines employed for target fishing. A total of 202 cell lines were excluded from these experiments because of having less than 20 compounds in any of the sensitive or resistant groups. The decision of a minimum of 20 compounds was based on Tropsha et al [27].

To train binary classification models, the two groups need to be balanced in the number of compounds that each one contains. A dataset was considered unbalanced if the majority group contained more than 1.2 times the number of compounds in the minority one. From the 758 cell lines, 213 were balanced whereas the remaining 545 ones underwent a data balancing procedure before modeling.

The data balancing protocol aimed at reducing the majority group to a similar number of compounds present in the minority class. The employed protocol relied in a hierarchical clustering procedure to retain the data diversity present in the initial majority group. For this, the chemical structures of the compounds in the majority group were coded using Morgan fingerprints of radius = 8 and 1024 bits. Then, the distance matrix between all pairs of compound belonging to the class to be reduced was computed employing the Tanimoto distance. The computed distance matrix was used as input to a tree-based clustering using the linkage function with the Ward’s minimum variance method. Clusters were defined using a cutoff height of 4.5.

If the number of clusters (Nc) was lower than the number of compounds in the minority group (Nm), then a number of compounds was selected from each cluster, proportional to the cluster size, until completing Nm samples. This was the case for 112 cell lines. On the other side, if Nc > Nm, then the majority group was re-clustered to obtain Nm clusters and the centroid of each one was selected for the reduced dataset. This procedure was applied to 443 cell lines.

The reduced datasets containing compounds coded with the Morgan fingerprints as described above were used to train Support Vector Machine (SVM) classification models with Radial Basis Function (RBF) kernels.

### Performance measures

Several performance metrics, previously reported in Peng et all [14] for target-fishing could be used. However, those metrics are not applicable to machine learning approaches. All those metrics are based on the amount of targets (cell lines) recovered for compounds. This means that the average is carried out across all compounds. The reason for this is that similarity-based strategies only consider sensitive compounds [14]. However, when compared to machine learning a different metric needs to be used because machine learning models where developed on the base of sensitive/resistant compounds per cell line. Therefore, the performance metrics are evaluated for each cell line and the average need to be computed across the cell lines space instead of compounds one. The metrics we used to compare both strategies are:

$$\langle TP\rangle =\frac{1}{Nc}\sum_{j=1}^{Nc}\frac{{TP}_{j}}{N_{j}^{AC}}$$ (Eq 1)

$$\langle TN\rangle =\frac{1}{Nc}\sum_{j=1}^{Nc}\frac{{TN}_{j}}{N_{j}^{RC}}$$ (Eq 2)

$$\langle ACU\rangle =\frac{1}{Nc}\sum_{j=1}^{Nc}\frac{{TP}_{j}+{TN}_{j}}{N_{j}^{AC}+N_{j}^{RC}}$$ (Eq 3)

Where Nc is the number of cell lines. TN and TP are the true-negative and true-positive of compounds per cell lines (j). Moreover, N_j_^AC^ and N_j_^RC^ are the number of compounds reported in the database as establishing a sensitive and resistant association respectively with cell line j.

### Validation strategies

The described performance metrics were computed for two different validation strategies: 1) the 10-fold cross-validation and 2) new compound with known sensitive/resistant annotations in the same cell lines space.

The SVM modeling was conducted using a 10-fold cross-validation setup. On each cross-validation cycle, 90% of the data was used to train a SVM model and the remaining 10% of it was employed as test set. Before training, the SVM C and γ parameters of the kernel were optimized by means of a grid search algorithm.

In order to get a comparative space between machine learning strategy and the similarity-based approach, the same compounds used in each 10-fold iterations (therefore the same compound in the partition) were evaluated with both prediction methods. All metrics (if not reported otherwise) calculated for machine learning model and the similarity-based method were computed with the initial database of 758 cell lines and 107195 compounds with 134565 sensitive and 145064 resistant LCLAs.

For the second type validation we carried on the same data treatment and filtering process already described for ChemBL v22 to ChemBL v25. With these new data we extracted the entire list of new compounds emerging from v22 to v25 and all their cell lines association (from the 758 cell lines): 11266 new compounds. 20898 sensitive pairs and 5237 resistant pairs (26135 total interactions).

### Web-service implementation

Two strategies are available as a web-service in; http://bioquimio.udla.edu.ec/cellfishing.

1. The CellFishing (similarity-based strategy). All 960 cell lines are included. In this section different Morgan’s Radius can be used.
2. The SVM models obtained in 758 cell lines.

## Supporting information

S1 Table List of cell lines and associated metadata. (XLSX)

S2 Table Sensitive and resistant compounds-cell lines pairs interaction database. (ZIP)

S3 Table Comparison between previous models and our current algorithm including cells and compounds diversity, source databases, modeling strategy and performance metric. (DOCX)
