# Supplementary material for: Cell fishing: A similarity based approach and machine learning strategy for multiple cell lines-compound sensitivity prediction
Source: PLoS One. 2019 Oct 7;14(10):e0223276. doi: 10.1371/journal.pone.0223276 (PMC6779297; doi:10.1371/journal.pone.0223276)
Supplement: S3 Table — (DOCX) [file pone.0223276.s003.docx]

**Supplementary Table.**

**Comparison with previous models used for compound-cell lines sensitivity prediction**

| **Method** | **Number of**  **Cell Lines** | **Number of**  **Compounds** | **Databases** | **Evaluation** | **Validation** | **Model** |
| --- | --- | --- | --- | --- | --- | --- |
| Lagunin et all [1] | 278 Cancer cell lines  27 normal cell lines | 59882 | ChemBL Database | accuracy of prediction (AUC) | 20-fold Cross-Validation | PASS model. Probabilistic approach. Require a training step for functions optimization. |
| Menden et all [2] | 608 Cancer cell lines | 111 | Cancer Genome Project [3] | R^2^ | 8-fold Cross-validation and External data | Neural network |
| Cortés-Ciriano et all [4] | 59 cell lines from NCI60 | 17142 | CellMiner database | R^2^ and RMSE | 10-fold Cross-validation | Random Forest and support vector machine |
| Ammad-ud-din et al [5] | 650 | 116 | Cancer Genome Project [3] | R^2^ and MSE | Cross-validation and test group | Bayesian Matrix Factorization |
| Zhang et al [6] | See notes |  | Use several datasets: Cancer Cell Line Encyclopedia [7] and Cancer Genome Project [3] | R, RMSE and NRMSE | Leave-One-Out cross-validation | Dual-Layer network |
| CellFishing ChemBLv 22 | 758 | 107195 | ChemBL Database | True Positive Rate, True negative Rate | 10-fold cross validation  Bootstrap  External | Tanimoto similarity  support vector machine |

Nota:

1) In Lagunin et al [1] the modelling Initially started with 943 human cell lines but the final model was only executed with those cell lines with AUC higher than 0.8 (278 cancer cell lines).

2) In the study of Zhang et al [6] several databases were used with different number of cell lines and compounds but the final number after integration is not clear in the publication. From Cancer Cell Line Encyclopedia 24 molecules and 504 cell lines while from Cancer Genome Project a total of 139 drugs and 653 or 707 cell lines.

3) The current model can be easily updated to 67900 compounds and 977 cell lines using the ChemBL v25 database.

**References**

1. Lagunin AA, Dubovskaja VI, Rudik A V., Pogodin P V., Druzhilovskiy DS, Gloriozova TA, et al. CLC-Pred: A freely available web-service for in silico prediction of human cell line cytotoxicity for drug-like compounds. Rishi A, editor. PLoS One. Public Library of Science; 2018;13: e0191838. doi:10.1371/journal.pone.0191838

2. Menden MP, Iorio F, Garnett M, McDermott U, Benes CH, Ballester PJ, et al. Machine Learning Prediction of Cancer Cell Sensitivity to Drugs Based on Genomic and Chemical Properties. Raghava GPS, editor. PLoS One. Public Library of Science; 2013;8: e61318. doi:10.1371/journal.pone.0061318

3. Garnett MJ, Edelman EJ, Heidorn SJ, Greenman CD, Dastur A, Lau KW, et al. Systematic identification of genomic markers of drug sensitivity in cancer cells. Nature. 2012;483: 570–575. doi:10.1038/nature11005

4. Cortés-Ciriano I, van Westen GJP, Bouvier G, Nilges M, Overington JP, Bender A, et al. Improved large-scale prediction of growth inhibition patterns using the NCI60 cancer cell line panel. Bioinformatics. Narnia; 2015;32: btv529. doi:10.1093/bioinformatics/btv529

5. Ammad-ud-din M, Georgii E, Gönen M, Laitinen T, Kallioniemi O, Wennerberg K, et al. Integrative and Personalized QSAR Analysis in Cancer by Kernelized Bayesian Matrix Factorization. J Chem Inf Model. American Chemical Society; 2014;54: 2347–2359. doi:10.1021/ci500152b

6. Zhang N, Wang H, Fang Y, Wang J, Zheng X, Liu XS. Predicting Anticancer Drug Responses Using a Dual-Layer Integrated Cell Line-Drug Network Model. Leslie CS, editor. PLOS Comput Biol. Public Library of Science; 2015;11: e1004498. doi:10.1371/journal.pcbi.1004498

7. Barretina J, Caponigro G, Stransky N, Venkatesan K, Margolin AA, Kim S, et al. The Cancer Cell Line Encyclopedia enables predictive modelling of anticancer drug sensitivity. Nature. 2012;483: 603–607. doi:10.1038/nature11003
